# Thrombocytosis in children and adolescents—classification, diagnostic approach, and clinical management

**DOI:** 10.1007/s00277-021-04485-0

**Published:** 2021-03-12

**Authors:** Clemens Stockklausner, C. M. Duffert, H. Cario, R. Knöfler, W. Streif, A. E. Kulozik, On behalf of THROMKID-Plus Studiengruppe der Gesellschaft für Thrombose- und Hämostaseforschung (GTH) and of Gesellschaft für Pädiatrische Onkologie und Hämatologie (GPOH)

**Affiliations:** 1 Department of Pediatric Oncology, Hematology and Immunology and Hopp Children’s Cancer Research Center (KiTZ), Heidelberg University and German Cancer Research Center (DKFZ), Heidelberg, Germany; 2 Department of Pediatrics, Garmisch-Partenkirchen Hospital, Auenstraße 6, 82467 Garmisch-Partenkirchen, Germany; 3 Department of Pediatrics, Heidelberg University, Heidelberg, Germany; 4 Department of Pediatrics and Adolescent Medicine, Ulm University Medical Center, Ulm, Germany; 5 Department of Pediatric Hematology and Oncology, Medical Faculty of Technical University, Dresden, Germany; 6 Department of Pediatrics, Medical University of Innsbruck, Innsbruck, Tirol Austria

**Keywords:** Pediatrics, Thrombocytosis, Hereditary thrombocytosis, Platelet disorders, Myeloproliferative neoplasms

## Abstract

Secondary thrombocytosis is a frequent secondary finding in childhood infection and inflammation. Primary hereditary thrombocytosis may be caused by germline mutations within the genes encoding key regulators of thrombopoiesis, i.e., thrombopoietin (*THPO*) and its receptor c-MPL (*MPL*) or the receptor’s effector kinase Januskinase2 (*JAK2*). Furthermore, somatic mutations in *JAK2*, *MPL*, and in the gene-encoding calreticulin (*CALR*) have been described to act as driver mutations within the so-called Philadelphia-negative myeloproliferative neoplasms (MPNs), namely essential thrombocythemia (ET), polycythemia vera (PV), and primary myelofibrosis (PMF). Increasing knowledge on the molecular mechanisms and on the clinical complications of these diseases is reflected by the WHO diagnostic criteria and European LeukemiaNet (ELN) recommendations on the management of adult MPN. However, data on childhood thrombocytosis are rare, and no consensus guidelines for pediatric thrombocytosis exist. Current literature has highlighted differences in the epidemiology and molecular pathogenesis of childhood thrombocytosis as compared to adults. Furthermore, age-dependent complications and pharmacological specificities suggest that recommendations tailored to the pediatric population are necessary in clinical practice. Here we summarize literature on classification, diagnostics, and clinical management of childhood thrombocytosis.

## Introduction

Recent research has allowed to better define the characteristics and pathogenesis of thrombocytosis allowing for substantial changes of its classification, risk stratification, and therapeutic approach. However, updated recommendations have so far centered on the adult population, and little data and guidance are available with regard to thrombocytosis in children and adolescents. Considerable differences have been described in the epidemiology and the clinical presentation of thrombocytosis in children as compared to adults. Transient thrombocytosis is a common finding in childhood infection and inflammatory processes. As Kucine et al. [1] pointed out this is especially true in young children, which may be caused either by the immaturity of the immune response in the young, by a higher incidence of infections or by more frequent iron deficiency in this group. However, acquired primary thrombocytosis (for definitions see below) as in Philadelphia-negative myeloproliferative neoplasms (MPNs) is significantly less frequent in children than in adults [2, 3]. Thus, a recent review described annual incidences of essential thrombocythemia (ET), one of the Philadelphia-negative myeloproliferative neoplasms (MPNs), ranging between 0.004 and 0.11 per 100,000 in children aged 0–16 years, while the meta-analysis in adults showed a pooled annual incidence rate of 1.03 per 100,000 (95% CI: 0.58–1.80) [4]. Besides essential thrombocythemia (ET), the Philadelphia-negative myeloproliferative neoplasms include polycythemia vera (PV) and primary myelofibrosis (PMF), and all three disorders are defined by excessive clonal proliferation in one or more of the hematopoietic lineages. Affected patients may suffer from thrombotic events, hemorrhage, microvascular disturbance, and leukemic or fibrotic transformation [5]. Recent research has elucidated the association between MPNs and somatic mutations in the genes *JAK 2* [6, 7], *MPL* [8], and *CALR* [9, 10]. In adults diagnosed with ET *JAK2-V617F* mutations are detected in approximately 55%, *CALR* mutations in 15–24%, and *MPL* mutations in 4% of cases, whereas around 20% of patients display no mutations in the three hotspot genes, which is referred to as “triple-negative” or “triple-wildtype” [11, 12]. However, a lower over-all incidence of these driver mutations was described in children diagnosed with ET [12]. Thus, in a group of 89 children with clinically diagnosed ET, Randi et al. found approximately 75% to be triple-negative and to display non-clonal disease [12]. Due to heterogeneity of adult and childhood ET, it has previously been emphasized that specific diagnostic criteria for MPNs in children are necessary [13]. Apart from genetic markers and clonality, the risk of complications, such as thromboembolic events in persistent thrombocytosis appears to be different in children. Thus, Teofili et al. found a significantly lower risk of thrombotic events for children with either familial or sporadic ET than in the respective adult comparison group [14]. Still, after a median follow-up of 6.3 years, Randi et al. reported three out of a group of 89 children (3.4%) with ET and *JAK2* mutations suffering from major thromboembolic complications, such as cerebral vein thrombosis and Budd-Chiari syndrome [12]. These observations highlight that thrombotic complications are relevant even in childhood but may require a distinct clinical approach. However, whereas guidelines for a risk-adapted therapy are available for adults affected by ET, there are no consensus recommendations tailored to the pediatric population [11]. The present paper therefore aimed to review the specific characteristics of thrombocytosis in children and adolescents with regard to classification, diagnostic approach, and therapy.

## Classification of pediatric thrombocytosis

**Fig. 1:**
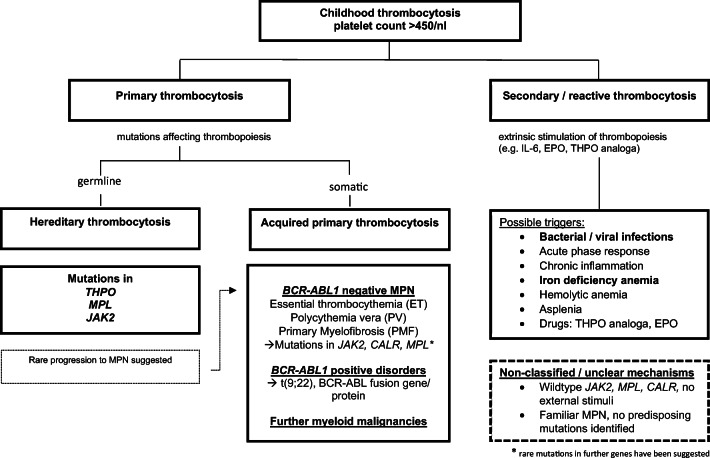
Classification of pediatric thrombocytosis

The suggested classification of childhood thrombocytosis is represented in Fig. 1. In accordance with previous classifications, thrombocytosis is defined as a platelet count exceeding 450 × 10^9^/l [1, 15]. Furthermore, platelet counts ranging from 450 to 700 × 10^9^/l can be described as *mild*, between 700 and 900 × 10^9^/l as *moderate*, between 900 and 1000 × 10^9^/l as *severe*, and those exceeding 1000 × 10^9^/l as *extreme* thrombocytosis [2]. Fu et al. pointed out that a higher threshold for the diagnosis of childhood ET might be helpful to minimize misdiagnosis of MPN in case of sustained secondary thrombocytosis [16]. Furthermore, age-specific alterations have to be taken into account at childhood age. Thus, further sources have proposed higher platelet levels to be considered physiological up to the age of 6 years [17]. The maximal cutoff of 650 × 10^9^/l was set at the age of 2 months with a gradual age-dependent decrease reaching adult values at the age of school entry [17]. A recent study reported sex-and age-dependent dynamics of platelet counts based on samples from 32,000 mostly white patients from a German population with similar results [18]. After an initial increase during the first months of life, platelet counts subsequently decreased and at school age, the 97.5th percentile approximately equalled the traditional cutoff of 450 × 10^9^/l [18]. Moreover, slight gender-specific differences were noted with higher platelet counts in females than in males [18]. These considerations should be kept in mind especially when examining early childhood thrombocytosis. In order to allow for a thorough clinical investigation and for reasons of clarity, a single cutoff of 450 × 10^9^/l was adopted in the algorithm and figures of the present review.

### Secondary or reactive thrombocytosis in children

As a first step, primary and secondary—or reactive—forms of thrombocytosis must be distinguished. In secondary thrombocytosis, elevated platelet levels result from an extrinsic process, such as acute or chronic inflammation, which stimulates megakaryocytopoiesis [2, 19]. Thrombopoiesis is regulated by the interplay between thrombopoietin (THPO) and its receptor c-Mpl [20, 21]. In secondary thrombocytosis, interleukin-6 (Il-6) constitutes an additional key mediator [22]. Secondary thrombocytosis is typically a transient phenomenon as it involves scaled-up platelet production without a permanent dysregulation of thrombopoiesis [23]. However, longer periods of several months and even years of secondary thrombocytosis may be observed especially in children, which constitutes a challenge to the diagnostic and classification process [16]. Secondary thrombocytosis is the most frequent form in both adults and in children and it is especially common in early childhood [2]. Besides bacterial or viral infection and iron-deficiency, triggers of secondary thrombocytosis include tissue damage, hemolytic anemia, asplenia, autoimmune disease, malignancies, and drug effects [2, 19]. Furthermore, transiently elevated platelet counts may be observed after episodes of severe bleeding or subsequent to myeloid recovery following chemotherapy [19]. In current classifications of pediatric erythrocytosis, germline mutations resulting in elevated erythropoietin (EPO) levels are classified as secondary erythrocytosis [24]. Still, in the present classification of pediatric thrombocytosis, germline mutations known to cause elevated THPO levels were grouped among the forms of primary thrombocytosis. This is in line with previous work on childhood thrombocytosis and allows to clearly distinguish between chronic and transient disorders, a feature that is among others pivotal to patients’ perception of the disease course [2, 23].

### Hereditary forms of primary thrombocytosis

In contrast to reactive forms, primary thrombocytosis is based on an intrinsic defect that entails dysregulation of the process of thrombopoiesis [23]. Typically, a mutation in genes associated with hematopoiesis can be detected [23]. Several authors have highlighted the importance of distinguishing between hereditary primary thrombocytosis and acquired forms with possible familial predisposition [2, 23]. Hereditary forms of primary thrombocytosis are caused by germline mutations within the genes encoding thrombopoietin (*THPO*), its receptor (*MPL*), and the receptor’s effector kinase Januskinase2 (*JAK2*) (see Table 1)*. MPL* mutations causing hereditary thrombocytosis most importantly include *MPL*-P106L [36], *MPL-*S505N [43], and *MPL*-W515R [46], as well as the polymorphism *MPL*-K39N termed MPL Baltimore [34]. Moreover, a recent report suggested germline *MPL*-R102P, that was formerly described as a homozygous, disease-causing mutation in congenital amegakaryocytic thrombocytopenia (CAMT), may paradoxically cause mild thrombocytosis when present in the heterozygous form [35, 60]. Of note, as opposed to hereditary thrombocytosis caused by *MPL* gain-of-function mutations, CAMT constitutes the clinical correlate to germline *MPL* loss-of-function mutations [60, 61], and association with aplastic anemia has been described in this context [62]. Interestingly, differences in both clinical phenotype and molecular characteristics have been described for thrombocytosis-inducing *MPL* mutations. Thus, the *MPL* gain-of-function mutation P106L was shown to confer constitutive, cytokine-independent receptor activity and elevated THPO serum levels despite impaired receptor glycosylation and surface expression [37, 63]. However, constitutively active receptors bearing mutations at codon 505 and 515 were glycosylated and expressed on the cell surface in cell models [43, 63]. Furthermore, apart from causing hereditary thrombocytosis when present in the germline, *MPL* mutations at codon 505 and 515 may occur as somatic mutations responsible for initiating MPNs including ET [8, 43, 44, 46]. The same phenomenon has been described for *JAK2* mutations at codon 617 [11, 42]; however, the *MPL* mutations P106L and K39N are not common in this context.

**Table 1:** Main germline mutations in hereditary thrombocytosis (HT)

Gene	Nucleotide change in THPO mRNA/MPL or JAK2 protein change	Mode of transmission if available	Described phenotypes	Comments	References
*THPO*					
	G516T, located in the 5-prime untranslated region (UTR)	Autosomal-dominant	HT with elevated THPO serum levels Distal limb defects	Loss of physiological translational inhibition. Referred to as G185T by Graziano et al.	Ghilardi et al. 1999 [25] Graziano et al. 2009 [26]
	c.13 + 1 G > C mutation in the splice donor of intron 3	Autosomal-dominant	HT with elevated THPO serum levels Vasomotor, thrombotic, and hemorrhagic symptoms AML Myelofibrosis Distal limb defects Multiple myeloma TMD	Loss of physiological translational inhibition.	Wiestner et al. 1998 [27] Liu et al. 2008 [28] Posthuma et al. 2010 [29] Stockklausner et al. 2012 [30] Houwing et al. 2015 [31]
	T > C transition at the splice donor of intron 2	Autosomal-dominant	HT with elevated THPO serum levels	Loss of inhibitory 5´-UTR sequence and exon 2 skipping.	Zhang et al. 2011 [32]
	Guanine deletion in the 5′-untranslated region	Autosomal-dominant	HT with elevated THPO serum levels	Location referred to as position 3252.	Kondo et al. 1998 [33]
	A to G mutation in intron 3	Autosomal-dominant	Elevated THPO serum levels		Reviewed by Teofili et al. 2011 [23]
*MPL*					
	K39N	Autosomal-dominant with incomplete penetrance	Isolated HT	Referred to as MPL Baltimore. Ca. 7% of the African American population are heterozygous for this polymorphism.	Moliterno et al. 2004 [34]
	R102P	Autosomal-dominant with incomplete penetrance	HT with elevated THPO serum levels	Described to cause rather mild thrombocytosis in heterozygous state (HT) and CAMT in homozygous state.	Bellanné-Chantelot et al. 2017 [35]
	P106L	Autosomal-recessive	HT with elevated THPO serum levels Hemorrhagic events in homozygous individuals	Ca. 6% of the Arabic population are carriers.	El-Harith et al. 2009 [36] Stockklausner et al. 2015 [37] Marri et al. 2013 [38]
	S505N	Autosomal-dominant	HT Thromboembolic events Myelofibrosis	Furthermore described as a somatic mutation in MPN (see Table 2).	Ding et al. 2004 [43] Beer et al. 2008 [44] Teofili et al. 2010 [45]
	W515R		HT	Furthermore described as a somatic mutation in MPN (see Table 2).	Vilaine et al. 2012 [46] Cabagnols et al. 2016 [47] Schnittger et al. 2008 [48]
*MPL* germline mutations requiring further investigation					
	R321W		ET Possibly HT	Described in heterozygous form in a patient with ET and clonal hematopoiesis. No family history of HT reported.	Milosevic Feenstra et al. 2016 [49]
	V285E		PMF Possibly HT	Described in heterozygous form in a patient with PMF and clonal hematopoiesis. No family history of HT reported.	Milosevic Feenstra et al. 2016 [49]
*JAK2*					
	R564Q	Autosomal-dominant	HT, possibly ET	Pseudokinase domain in exon 13 affected. Clonality assays not entirely conclusive.	Etheridge et al. 2014 [50]
	H608N	Likely autosomal-dominant with incomplete penetrance	HT	Pseudokinase domain affected.	Rumi et al. 2014 [51]
	L611S		Single case description, likely HT	No family history of HT in the described pediatric case, considered as de novo mutation. Furthermore described as somatic mutation in ALL, PV.	Aral et al. 2018 [52]
	V617I	Autosomal-dominant	HT and ET, Vascular events in 3 patients aged > 40 years	Furthermore described as somatic mutation (see Table 2). Pseudokinase domain affected. Located in Exon 14.	Mead et al. 2012 [42] Beucher et al. 2019 [41]
	V625F		ET/HT	Gain-of-function effect demonstrated. Clonality analysis not conclusive. No family history reported.	Milosevic Feenstra et al. 2016 [49]
	S755R/R938Q in cis		HT	Pseudokinase domain affected by S755R, kinase domain affected by R938Q.	Marty et al. 2014 [53]
	T875N		Clinical diagnosis of ET, likely HT. One family member with cerebral infarction at age 51, no mutation status available.	Family history of HT, however no mutation status or clonality assessment available. Located in exon 18.	Yoshimitsu et al. 2019 [54]
	R867Q		HT, progression to PV suggested	Kinase domain affected. Furthermore described as somatic mutation in ALL.	Marty et al. 2014 [53] Maie et al. 2018 [55]
	R938Q		HT	Furthermore described as somatic mutation in a case of pediatric ALL and in cis with S755R (see above).	Sadras et al. 2017 [56] Marty et al. 2014 [53]
*JAK2* germline variation predisposing for MPN					
	GGCC or 46/1 haplotype		Predisposition to the development of MPN including ET	Combination of SNPs including the 3′region of *JAK2.*	Kilpivaara et al. 2009 [57] Jones et al. 2009 [58] Olcaydu et al. 2011 [59]
*JAK2* mutations requiring further investigation					
	G335D		Association with ET HT not described	Described in heterozygous form in an ET patient. No gain-of-function effect detected.	Milosevic Feenstra et al. 2016 [49]
	G571S		Association with ET HT not described	Described in heterozygous form in an ET patient. No gain-of-function effect detected.	Milosevic Feenstra et al. 2016 [49]
	F556V		HT/ET	Mutation status not specified, possibly germline mutation conferring HT. Gain-of-function effect demonstrated.	Milosevic Feenstra et al. 2016 [49]
	N1108S		HT/ET	Described as rare polymorphism but also as a germline mutation in a case of ET.	Cabagnols et al. 2016 [47]

*ALL*, acute lymphatic leukemia; *AML*, acute myeloid leukemia; *ET*, essential thrombocythemia; *CAMT*, congenital amegakaryocytic thrombocytopenia; *PMF*, primary myelofibrosis; *SNP*, single nucleotide polymorphism; *TMD*, transient myeloproliferative disease

From a clinical point of view, hereditary primary thrombocytosis has mostly been regarded as a benign disorder with polyclonal hematopoiesis without significant risk of thromboembolic complications [64]. However, studies by Teofili et al. demonstrated an increased risk of thromboembolic events and fibrotic transformation in patients harboring germline *MPL*-S505N [45]. Interestingly, the same mutation was described in the context of MPN when present somatically in hematopoietic cells [44]. Furthermore, bleeding as a consequence of extreme thrombocytosis with acquired von Willebrand syndrome (AvWS) was reported in three siblings affected by *MPL*-P106L [38].

With regard to *THPO*, several mutations have been described to reduce the physiological inhibition of THPO-mRNA translation leading to higher serum levels of the hormone and consequently thrombocytosis [25, 27, 28, 32, 33, 65]*.* Conversely, it was described that *THPO* loss-of-function mutations may cause thrombocytopenia, aplastic anemia [66], and bone marrow failure unresponsive to bone marrow transplant [67]. Clinically, hereditary primary thrombocytosis based on *THPO* germline mutations has been described to be associated with vasomotor, hemorrhagic, and thrombotic symptoms [28]. Furthermore, association with distal limb defects [26, 30], multiple myeloma, fibrotic, and leukemic transformation, respectively, have been reported in families carrying THPO germline mutations [29, 30]. Moreover, transient myeloproliferative disease was reported in two infants of a family affected by hereditary thrombocytosis caused by a mutation in intron 3 of the THPO mRNA [31]. Similarly, a transient myeloproliferative disorder resembling chronic myeloid leukemia (CML) has been described in an infant with an inherited THPO mutation [68].

Regarding *JAK2,* both germline mutations at the hot-spot codon 617 and at further loci affecting its kinase and pseudokinase domains have been described to cause congenitally elevated platelet counts [64]. Vascular complications were reported in three > 40-year old members of a family with germline *JAK2*-V617I [42]. Moreover, a first-degree relative of a patient with germline JAK2-T875N and chronic thrombocytosis was reported to have suffered from cerebral infarction at 51 years [54].

The relationship between hereditary thrombocytosis and MPN remains poorly understood. Both *MPL* and *JAK2* mutations have been described to occur in patients with hereditary thrombocytosis and—in somatic form—in MPNs. Single case reports have postulated progression to MPN in patients with germline mutations that are known to cause hereditary thrombocytosis, such as development of PV in the presence of *JAK2*-R867Q [54, 55]. Because of the central role of somatic *JAK2* mutations in MPNs, it seems likely that germline mutations may predispose for the development of MPNs [64]. Of note, in most families with clustered MPNs, often referred to as “familial MPNs,” no causative mutations have been identified so far [64, 69], and the risk of developing MPN in hereditary thrombocytosis—independent of the causative mutation—is not well-defined. However, although sufficient prospective data are currently lacking, case reports suggest that clinical monitoring and possibly prophylactic treatment in the presence of hereditary thrombocytosis may be necessary.

### Acquired forms of primary thrombocytosis in children and adults

**Fig. 2:**
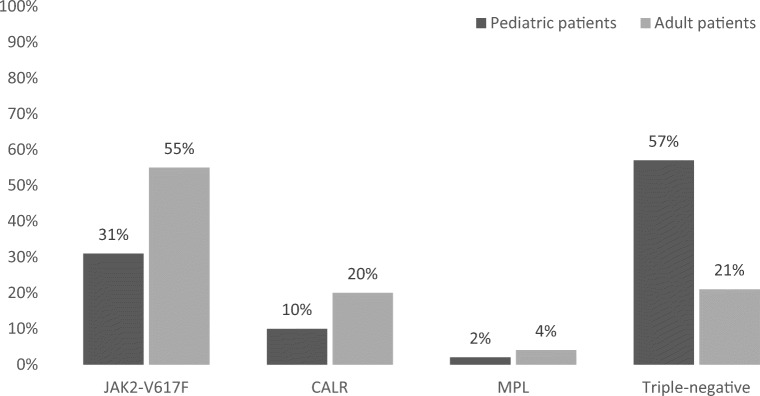
Frequencies of the main driver mutations in essential thrombocythemia in children compared to adults. Data on children according to Ianotto et al. [78]. Data on adults according to Tefferi et Barbui [11]

Acquired primary thrombocytosis is marked by somatic mutations and occurs in several myeloid malignancies that are more commonly found in adults than in children. Therefore, most of the current knowledge on their pathogenesis and on their clinical management has been derived from studies in adults. Myeloid malignancies that may involve acquired primary thrombocytosis include myeloproliferative (MPNs) and myelodysplastic neoplasms (MDS), such as Philadelphia-positive chronic myeloid leukemia (CML), the Philadelphia-negative neoplasms ET, PV, and PMF, MPN/MDS with ring sideroblasts and thrombocytosis (RARS-T) or MDS with isolated del(5q) syndrome [23, 70]. The Philadelphia-positive disorders are marked by a translocation of chromosome 9 and 22 generating the BCR-ABL1 fusion gene and protein. Therefore, screening for *BCR-ABL* represents a necessary diagnostic step in primary thrombocytosis. Within the group of Philadelphia-negative myeloproliferative neoplasms, excessive platelet counts are a central feature of ET. In PV, erythrocytosis constitutes the main abnormality, and an elevated platelet count alone is not a major criterium for diagnosis of PMF or pre-PMF [70]. Drivers of the Ph-negative MPNs include mutations of *JAK2*, most importantly *JAK2*-V617F [6, 7] in exon 14 (see Table 2), exon 9 mutations of *CALR* [9, 10] (see Table 3), and exon 10 *MPL* mutations especially at codon 515 [8, 48] (see Table 4) (Fig. 2). Furthermore, a number of rare *MPL* mutations have been detected in mostly single cases of adult ET or other MPNs [49] (Table 4). The clinical picture of pediatric ET was furthermore described in the presence of *MPL*-Y252H [72]. Rare *JAK2* mutations in exon 12 and in exon 14 have been described in PV [11] but are not commonly found in adult ET. Besides the Philadelphia-negative MPN, somatic mutations in *MPL* have been described in the context of other pediatric hematological disorders including idiopathic-acquired aplastic anemia, myelodysplastic syndrome (MDS) [79], and acute megakaryoblastic leukemia (AMKL) in childhood [80]. The same applies to somatic mutations in *JAK2* that have been reported in a wide spectrum of diseases, such as acute lymphoblastic leukemia in pediatric patients with trisomy 21 [39], acute myeloid leukemia (AML) in adulthood [40], and myelodysplastic/myeloproliferative neoplasms (MDS/MPN) with ring sideroblasts and thrombocytosis (MPN/MDS-RS-T) [81]. Somatic *THPO* gain-of-function mutations were reported to be extremely rare in the context of sporadic myeloproliferative malignancies [31]. One case of a 7-year-old patient suffering from AML that carried a *THPO* mutation was described; however, in the reported case, presence of the mutation in the germline could not be excluded [31].

**Table 2:** Main somatic *JAK2* mutations in essential thrombocythemia (ET)

Exon	Protein change	Described phenotypes	Comments	References
14	V617F	ET, PMF, PV, further hematological malignancies such as AML, ALL	Most common somatic mutation in MPN, detected in ca. 55% of cases of adult ET. Affecting the pseudokinase domain.	Kralovics et al. 2005 [7] Bercovich et al. 2008 [39] Lee et al. 2006 [40]
14	V617I	MPN, ET	Affecting the pseudokinase domain. Furthermore described as germline mutation causing HT (see Table 1).	Beucher et al. 2019 [41] Mead et al. 2012 [42]

*ALL* acute lymphatic leukemia. *AML*, acute myeloid leukemia. *ET*, essential thrombocythemia; *HT*, hereditary thrombocytosis; *PMF*, primary myelofibrosis; *MPN*, myeloproliferative neoplasm

Multiple further somatic mutations in *JAK2* have been described in MPN other than ET or in MPN without further specification

**Table 3:** Somatic *CALR* exon 9 mutations in essential thrombocythemia (ET)

Type of mutation	Protein change	Described phenotypes	Comments	References
> 30 insertions or deletions causing a frameshift leading to premature termination and a changed C-terminal		ET, PMF, RARS-T, MDS, CML, atypical CML	Found in 15–24% of cases of adult ET	Klampfl et al. 2013 [9] Nangalia et al. 2013 [10] Tefferi et al. 2019 [11]
	p.L367fs*46	ET PMF	Ca. 45–53% of *CALR* mutations in MPN Referred to as *CALR* type 1 mutation	Klampfl et al. 2013 [9] Nangalia et al. 2013 [10]
	p.K385fs*47	ET PMF	Ca. 32–41% of *CALR* mutations in MPN Referred to as *CALR* type 2 mutation	Klampfl et al. 2013 [9] Nangalia et al. 2013 [10]

*ET*, essential thrombocythemia; *CALR*, calreticulin; *PMF*, primary myelofibrosis; *RARS-T*, refractory anemia with ring sideroblasts associated with marked thrombocytosis; *CML*, chronic myeloid leukemia

**Table 4:** Main somatic *MPL* mutations in essential thrombocythemia (ET)

Exon	Protein change	Described phenotypes	Comments	References
3–9				
	T119I	ET	Exon 3.	Milosevic Feenstra et al. 2016 [49]
	*F126fs*	ET	Mutation affecting the extracellular domain. No bone marrow examination in the case report.	Elsayed et al. 2019 [71]
	S204F	ET, PMF	Exon 3.	Milosevic Feenstra et al. 2016 [49] Cabagnols et al. 2016 [47]
	S204P	PMF, ET	Exon 4.	Milosevic Feenstra et al. 2016 [49] Cabagnols et al. 2016 [47]
	E230G	ET	Exon 5.	Milosevic Feenstra et al. 2016 [49]
	Y252H	ET	Mutation affecting the extracellular domain. Pediatric case with clonal hematopoiesis described.	Lambert et al. 2012 [72] Elsayed et al. 2019 [71]
10				
		ET	The most common *MPL* mutations in ET are located in exon 10, especially codon 515. Found in ca. 4% of cases of adult ET.	Tefferi et al. 2019 [11]
	A497-L498ins4	ET		Xie et al. 2019 [73]
	V501A	ET, PMF	Occurrence in combination with MPL-W515L/R.	Pietra et al. 2011 [74]
	S505C	ET	Occurrence in combination with MPL-W515L. Classified as likely pathogenic.	Pietra et al. 2011 [74]
	S505N	ET, PMF, HT	Furthermore occurrence as germline mutation causing HT.	Ding et al. 2004 [43] Beer et al. 2008 [44]
	W515K	ET, MMM		Pardanani et al. 2006 [8]
	W515L	ET, MMM		Pikman et al. 2006 [75] Pardanani et al. 2006 [8]
	W515A	ET		Schnittger et al. 2008 [48]
	W515R	ET, PMF	Furthermore described as germline mutation causing HT (see Table 1).	Cabagnols et al. 2016 [47] Schnittger et al. 2008 [48] Vilaine et al. 2012 [46]
12				
	Y591N	ET		Cabagnols et al. 2016 [47]
	Y591D	ET, PV	Both in ET and in PV described in the presence with further mutations.	Milosevic Feenstra et al. 2016 [49]
Mutations requiring further investigation				
	P70L	ET	No gain-of-function effect confirmed.	Chang et al. 2018 [76]
	V501A	*JAK2*-negative *MPN*	Occurrence in combination with MPL-S505N described.	Ma et al. 2011 [77]
	V507I	*JAK2*-negative *MPN*		Ma et al. 2011 [77]
	W515G	*JAK2*-negative *MPN*	Exon 10 mutation.	Ma et al. 2011 [77]
	W515S	*JAK2*-negative *MPN*	Exon 10 mutation.	Ma et al. 2011 [77]
	W515R Q516E	ET	Double point mutation. Model predicted gain-of-function for Q516E.	Xie et al. 2019 [73]
	W515-P518 del/ins KT	*JAK2*-negative *MPN*	Exon 10 mutation.	Ma et al. 2011 [77]

*ALL*, acute lymphatic leukemia; *AML*, acute myeloid leukemia; *ET*, essential thrombocythemia; *HT*, hereditary thrombocytosis; *MMM*, myelofibrosis with myeloid metaplasia; *MPN*, myeloproliferative neoplasm; *PMF*. primary myelofibrosis

In addition to *JAK2*, *MPL*, and *CALR*, rare variants involving regulators of JAK2, such as LNK [82], and a number of further loci including *TET2* [83], *IDH1/IDH2* [84], *ASXL1* [85], *CBL* [86], *IKZF1* [87], *SF3B1*, *U2AF1*, *TP53*, *EZH2* have been suggested to be associated with the course of the disease and in some cases to predispose for the development of MPNs [1, 69, 88]. As mentioned above, familial clustering of MPNs has been frequently described, and first-degree relatives of MPN patients were estimated to have a five- to sevenfolds increased risk than the average population [89]. However, the underlying pathogenesis remains insufficiently explained [23, 64]. As single cases of *JAK2* germline mutations have been described in the context of ET [49] and PV [55], it may be discussed whether specific germline mutations in *JAK2* but also other genes including *MPL*, *CALR*, or *THPO* may predispose carriers for MPN. Since the occurrence of both, different forms of MPNs and different driver mutations have been described within single families, it has been suggested that specific inherited traits may predispose for acquiring somatic mutations, including the described driver mutations [59]. Thus, familial clustering was, for example, postulated to be triggered by specific germline variants in *JAK2* [57] or other genes such as *ATG2B*, *GSKIP* [69]. Furthermore, a specific *JAK2* haplotype (46/1 haplotype) was suggested to predispose individuals for MPN [58, 69]. Still, in summary, the main fraction of familial clustering of MPN remains insufficiently understood.

As mentioned before, approximately 55% of adults diagnosed with ET display *JAK2* mutations, 15–24% *CALR* mutations, and 4% *MPL* mutations, whereas around 20% are triple-negative [11]. Studies with a focus on adult triple-negative ET indicated that some patients may carry very rare mutations in *MPL* or *JAK2* not detected in routine mutation screening [47, 49]. Furthermore, several patients were found to have polyclonal hematopoiesis and were probably not affected by a true MPN [47]. In children, the proportion of triple-negative and therefore molecularly unexplained patients with clinically diagnosed ET seems to be even higher. Thus, among a group of 89 children with clinically diagnosed ET described by Randi et al. around 75% were found to be triple-negative [12]. Furthermore, a recent literature review described that in 57% of identified patients with childhood ET (*n* = 396), none of the three known driver mutations were detected [78]. Randi et al. found that among the remaining 25% of pediatric patients with ET, the distribution of the established main driver mutations resembled the ratio observed in adults [12]. While Teofili et al. did not detect significant differences in the occurrence of clonal disease in sporadic childhood ET when compared to adults, Randi et al. reported that nearly 75% of children diagnosed with ET displayed non-clonal disease [12, 14]. The authors described that within this subgroup, some children suffered from clinical complications typical of ET whereas others remained largely asymptomatic [12]. Since ET is per se defined as clonal disease, this subgroup of apparent ET but absence of clonality may either represent an additional clinical entity in pediatric thrombocytosis with a specific disease-causing mechanism or the described patients may have been affected by prolonged secondary thrombocytosis. This is important as in both cases, a clinical approach distinct from that in true ET would be required, e.g.,—in case of secondary thrombocytosis—close monitoring rather than invasive diagnostics and therapy [12].

These findings highlight that there remains a fraction of patients with sustained thrombocytosis whose disease is difficult to classify. The increasing use of genomic and epigenomic analyses will likely contribute to our further understanding of disease-causing mechanisms in childhood thrombocytosis.

## Diagnostic approach to childhood thrombocytosis

**Fig. 3:**
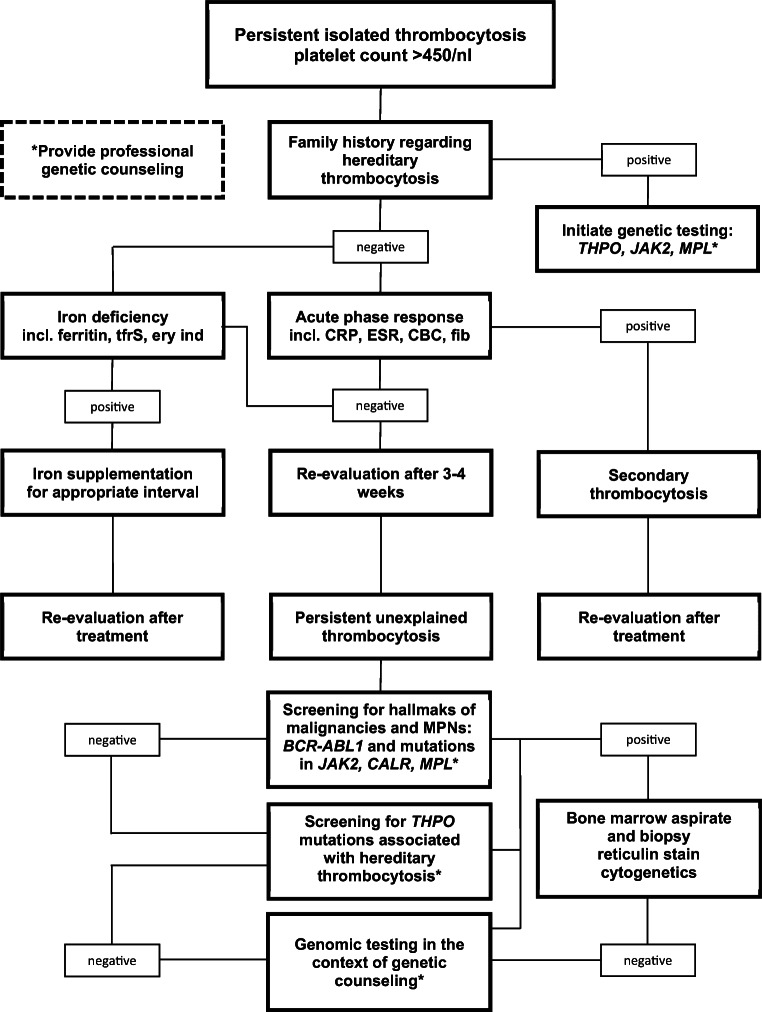
Diagnostic algorithm to childhood thrombocytosis. Modified after Harrison et al. [90] and Kucine et al. [1]. ABL, gene-encoding abelson kinase; BCR, gene named breakpoint cluster region; CRP, C-reactive protein; CALR, gene-encoding calreticulin; ESR, erythrocyte sedimentation rate; CBC, complete blood count; Fib, fibrinogen; Incl., including; *JAK2*, gene-encoding Januskinase2; MPN, myeloproliferative neoplasm. *THPO*, gene-encoding thrombopoietin; TfrS, transferrin saturation; Ery ind, erythrocyte indices

Because of the differences in the epidemiology and clinical presentation of thrombocytosis in childhood when compared to adults, an algorithm specifically adapted to the pediatric population is clearly required for clinical practice. Both Harrison et al. and Kucine et al. described diagnostic work-ups for thrombocytosis, and childhood thrombocytosis respectively [1, 90]. With growing awareness of germline mutations as causes of hereditary thrombocytosis, Teofili et al. highlighted the importance of excluding hereditary thrombocytosis before subjecting children to inappropriate invasive diagnostics [13]. Figure 3 presents a diagnostic algorithm accounting for early detection of hereditary forms of childhood thrombocytosis. Limitations arise from the lack of clinical prospective data especially on primary thrombocytosis in children.

In case of sustained, isolated thrombocytosis, defined as a platelet count above 450 × 10^9^/l, a thorough medical history and physical examination addressing the family history, and causes of secondary thrombocytosis (see Fig. 1) are essential. Regarding the distinction between transient and sustained thrombocytosis, Kucine et al. proposed an interval of 3–4 weeks before re-evaluating platelet counts in case of unexplained thrombocytosis [1]. In case of a family history indicating hereditary thrombocytosis and in the absence of causes of secondary thrombocytosis, it may be appropriate to directly initiate genetic diagnostics by sequencing the most commonly affected genes following professional genetic counseling (see Table 1). This approach is in line with suggestions by Teofili et al. who highlighted the importance of early detection of hereditary thrombocytosis [13]. Furthermore, when suspecting hereditary thrombocytosis, affected adult relatives may be analyzed first in order to minimize diagnostic procedures in children [24]. In all other cases, laboratory diagnostics should first concentrate on excluding secondary thrombocytosis thus including markers of inflammation such as C-reactive-protein (CRP), erythrocyte sedimentation rate (ESR), fibrinogen, as well as a complete blood count (CBC) with a differential white blood count (WBC). Furthermore, determining erythrocyte indices, red cell distribution width (RDW), ferritin, and transferrin saturation is essential to exclude iron-deficiency anemia accompanied by thrombocytosis. If iron deficiency is detected together with thrombocytosis in an otherwise asymptomatic child, iron supplementation should be initiated according to current guidelines and platelet counts re-evaluated after correction of the iron deficiency. Similarly, if secondary thrombocytosis caused by an acute phase reaction is suspected, platelet counts should be reassessed after inflammation has subsided*.* Of note, elevated markers of inflammation may require further investigation regarding possible underlying causes, and the diagnostic assessment should also consider autoimmune disease.

If secondary thrombocytosis is excluded (see Fig. 1), primary thrombocytosis has to be considered. Of note, if sustained thrombocytosis is not isolated but paralleled by further changes in the blood count, a wider range of hematological disorders have to be considered from the start. Thus, in case of sustained thrombocytosis combined with an increase of the red cell mass, i.e., hemoglobin and hematocrit levels exceeding the age-related reference ranges, PV has to be excluded.

In case of suspected primary thrombocytosis, genetic testing accompanied by professional genetic counseling should address hallmarks of myeloid malignancies such as *BCR-ABL1* and mutations associated with MPNs, that is *JAK2*, *MPL*, and *CALR* (for the respective mutations see Tables 2, 3, and 4). Although the number of mutations causing primary thrombocytosis has recently increased, a stepwise approach first targeting the three hot-spot genes *JAK2*, *MPL*, and *CALR* besides screening for *BCR-ABL1* seems to be justified. Still, whereas testing for *JAK2*-V617F, exon 9 mutations in *CALR* and exon 10 mutations in *MPL* should identify more than 75% of ET cases in adults; this proportion is likely to be substantially lower in children and adolescents, and a more extensive diagnostic testing may be necessary [11, 12]. Thus, extending the genetic testing especially to further exons of *MPL* and *JAK2* may be required. If a somatic mutation indicative of myeloid malignancies or myeloproliferative disease is detected, bone marrow examination including reticulin staining and cytogenetics will allow to confirm the diagnosis of MPN. As underlined by Tefferi et al., bone marrow morphology is required to distinguish between ET and prefibotic-PMF [11]. If the diagnosis of pediatric ET is established, screening for additional thrombophilic risk factors (e.g., protein C-, S- and antithrombin deficiency, factor V Leiden, and prothrombin mutation), risk factors for hemorrhage such as presence of acquired von Willebrand syndrome (AvWS), as well as for cardiovascular risk factors will be required for decision-making on clinical management [1].

If the first genetic screening remains without positive findings, testing for *THPO* mutations in the 5′UTR region in order to exclude further causes of hereditary thrombocytosis is recommended. Nelson et al. suggested that prior to genetic testing, measuring THPO serum levels may support the diagnostic process as both HT caused by *THPO* mutations and by *MPL*-P106L mutations have been described to entail markedly elevated THPO levels [37, 91]. However, measurement of THPO levels is not commonly offered by laboratories, and commercially available kits for measuring THPO are usually licensed for research use but not for diagnostic procedures. Therefore, measuring THPO serum levels may prove challenging in daily clinical practice. In case of suspected HT, family studies and the analysis of non-hematopoietic cells such as fibroblasts can be employed to distinguish between somatic and germline mutations. Furthermore, when hereditary thrombocytosis is detected, bone marrow examination should be considered in order to exclude fibrotic or possibly malignant transformation.

As an alternative to this two-step approach, screening for *BCR-ABL1* and a genetic panel addressing mutations in *JAK2*, *MPL*, *CALR*, as well as *THPO* may be initiated from the start paralleled by professional genetic counseling. Finally, if genetic testing remains without findings and thrombocytosis persists, bone marrow examination for further exploration should be considered, and genomic testing may be proposed to the family in the context of professional genetic counseling.

## Management of childhood thrombocytosis

### What are the main complications of chronic thrombocytosis?

There are only limited data on how childhood thrombocytosis should be monitored and treated and in how far recommendations for adults can be applied to children and adolescents [90]. The occurrence of thromboembolic complications, one of the major concerns in persistent thrombocytosis, is far more common in primary than in secondary thrombocytosis [92]. Therefore, in the absence of additional risk factors, secondary thrombocytosis in otherwise healthy children does not warrant specific treatment, and therapy is directed at the underlying causes. In adult ET and PV, risk-adapted therapy seeks to limit thromboembolic complications, to prevent hemorrhage from acquired von Willebrand syndrome (AvWS) and to control microcirculatory symptoms such as headaches or neurological symptoms such as paresthesia [11]. Tefferi and Barbui described the risk of leukemic transformation after 20 years of the diagnosis of thrombocytosis to amount to 5% in ET and less than 10% in PV [11]. Secondary myelofibrosis was estimated to be slightly more common [11]. In young patients, malignant transformation is rarely observed. A recent literature review that analyzed 396 patients with ET and 75 patients with PV that were < 20 years of age, respectively, found fibrotic transformation in 2% of patients and no development of acute leukemia. However, follow-up was limited to a median of 50 months [78]. The risk of thrombotic events in adult ET has been estimated between 1.5 and 2.5% per patient-year. Age over 60 years, presence of the driver mutation *JAK2*-V617F, presence of cardiovascular risk factors, and history of thrombosis were identified to constitute independent risk factors for arterial thrombosis [93]. Considering that the occurrence of thromboembolic events is age-dependent and that cardiovascular risk factors are less prevalent in children and adolescents, a lower incidence for thromboembolic events may be expected in patients with primary thrombocytosis in this age group. Indeed, a study led by Teofili et al. found a significantly lower risk of thrombotic events in children with either familial or sporadic essential thrombocythemia than in the respective adult comparison group [14]. Among the 396 patients with ET in the age group below 20 years that were analyzed by Ianotto et al., thrombotic complications were reported in only 3.8% [78]. However, as mentioned above, the authors emphasized that the time of follow-up in the identified cases was limited to a median follow-up of 50 months [78]. Furthermore, given the high percentage of triple-negative patients and the difficulties in correctly diagnosing MPN in children, some patients might have been misdiagnosed, and complications therefore underestimated. When considering the risk of thrombocytosis in children with ET, it must also be noted that the thrombotic events reported in children tended to be clinically severe. Thus, Randi et al. reported three out of the 89 (3.4%) children with a clinical diagnosis of ET and detectable *JAK2*-V617F mutation suffering from Budd Chiari syndrome (two cases) and cerebral vein thrombosis (one case), equaling 13% in the subgroup of children with clonal disease [12].

### What can be learned from clinical management of adult ET?

Guidelines for risk-adapted therapy in adult ET by the European LeukemiaNet (ELN) recommend assessing patients’ thrombotic risk by using the International Prognostic Score for Thrombosis in ET (IPSET) [94]. By means of this tool, patients are assigned to a low-, intermediate-, or high-risk group based on the criteria of age over 60 years, presence of the driver mutation *JAK2*-V617F, presence of cardiovascular risk factors, and history of thrombosis [94]. Tefferi and Barbui suggested to monitor patients with ET aged below 60 years, without cardiovascular risk factors and without *JAK2* mutations [11]. Anti-platelet therapy with acetylsalicylic acid (ASA) is recommended in patients > 60 years or in the presence of either the driver mutation *JAK2*-V617F or uncontrolled cardiovascular risk factors or a history of thrombosis provided there are no risk factors for hemorrhage [11, 94]. Depending on the individual risk factors, ASA intake is recommended once to twice daily [11]. In case of a history of venous thrombosis, anti-platelet therapy usually does not suffice but systemic anticoagulation is necessary and may be combined with ASS in the presence of a *JAK2* mutation or cardiovascular risk factors [11]. Regarding the risk of bleeding in ET, it has been suggested that especially patients with platelet counts exceeding 1000 × 10^9^/l, thus presenting with extreme thrombocytosis, may be prone to bleeding episodes caused by acquired von Willebrand syndrome (AvWS) [95]. Tefferi et Barbui therefore recommended to measure von Willebrand factor (vWF) antigen and a parameter reflecting vWF function (i.e., vWF or ristocetin cofactor activity) in patients with either extreme thrombocytosis or abnormal bleeding irrespective of platelet counts [11]. In case of presence of AvWS, therapy with ASA in low-risk patients should be paused [11, 70]. In addition, cytoreductive therapy was recommended in patients aged 60 years and above [94] although not judged mandatory by Barbui et Tefferi in the absence of cardiovascular risk factors, a history of thrombosis, and *JAK2* mutations [11]. Patients aged 60 years and above with either cardiovascular risk factors or *JAK2* mutations, as well as any patients presenting with a history of thrombosis, should receive cytoreductive treatment [11]. Furthermore, the ELN recommendations include cytoreductive therapy in case of platelet counts exceeding 1500 × 10^9^/l [94] or major bleeding episodes [94]. Additionally, cytoreductive treatment may be considered in order to control systemic symptoms or those caused by myeloproliferation [94].

As to the choice of cytoreductive agent, *hydroxyurea* was recommended as first-line therapy in adults [11, 94]. Its use in younger patients was controversially discussed in the past due to potential leukemogenicity [11, 96]. However, Barbui et Tefferi emphasized that current evidence including long-term therapy in children with sickle-cell disease does not support these concerns [11]. Several agents have been suggested as second-line therapy in adults. *Interferon-alpha* (IFN-alpha) has been suggested as a safe treatment option in pregnant women and in younger patients [11, 94, 96]. It has been shown to effectively reduce platelet counts as well as *JAK2*-V617F burden and to alleviate symptoms [97, 98]. However, common side effects include influenza-like symptoms as well as neuro-psychiatric symptoms, such as depression and irritability, and may interfere with compliance to treatment [99]. Still, the availability of new pegylated IFN preparations has already allowed to extend the interval between injections and to improve drug tolerance. *Anagrelide* constitutes a further non-leukemogenic treatment option and specifically reduces the platelet count [100]. Thus, analyses from the EXELS study suggested that anagrelide seems to be the safer alternative regarding the development of acute leukemia compared to hydroxyurea and further cytoreductive agents, which would constitute a definite advantage for cytoreductive therapy in children [101, 102]. Regarding its efficacy, the ANAHYDRET study, a prospective randomized trial, found non-inferiority of anagrelide compared to hydroxyurea [103], and several studies even suggested a lower risk of venous thromboembolism [101, 104]. However, both the randomized PT-1 trial [104] and the observational EXELS study [101] suggested a higher risk of fibrotic transformation, hemorrhagic events, and arterial embolism in adults treated with anagrelide as compared to those treated with hydroxyurea or other agents, respectively. For this reason, adult guidelines have so far favored the use of hydroxyurea over anagrelide as first-line therapy. Finally, due to concerns about leukemogenicity, the use of *busulfan* is restricted to elderly adults and not recommended as first-line therapy [11, 96].

### When to consider anti-platelet therapy in children?

Because of the potentially increased risk of complications such as Reye syndrome [96] in children and adolescents, as well as the probably more benign course of disease, the need of cautious decision-making has been emphasized repeatedly [90, 96]. With regard to Reye syndrome, epidemiological data and information on the exact risk of this complication under therapy with ASA are scarce. Low dosage of ASA as in therapy of adult MPN may reduce but not completely eliminate the risk of Reye syndrome, especially in the age group below 12 years [90, 96]. However, Reye syndrome is in general described as a rare disorder. Thus, one analysis estimated its annual incidence in the United States (US) to have been between 0.2 and 1.1 cases per million in the population < 18 years during the period from 1991 to 1994 [105]. In line with a restrictive treatment approach to chronic thrombocytosis, Giona et al. proposed that any prophylactic treatment should be avoided at infant age [106]. Kucine et al. recommended clinical monitoring including determination of platelet counts in intervals of 3 to 6 months for asymptomatic pediatric patients with ET in the absence of additional thrombophilic risk factors [1]. This approach corresponds to the recommendations for adults aged below 60 without cardiovascular risk factors and without *JAK2* mutations [11]. While emphasizing that the suggested treatment algorithm would require validation via analysis of large patient collectives, Kucine et al. divided children with ET into a low- and high-risk category [1]. Those with milder symptoms including hepatosplenomegaly, microvascular disturbances, such as headaches or erythromelalgia, as well as asymptomatic children with further thrombophilic or cardiovascular risk factors in addition to ET were considered low-risk [1]. Low-dose ASA with careful monitoring for bleeding complications or potential Reye syndrome was suggested as a reasonable therapeutic approach for this group [1]. Regarding the dosage, Harrison et al. suggested using 2–3 mg/kg of ASA with a maximum of 75 mg per day in anti-platelet therapy in childhood ET [90]. Furthermore, the American College of Chest Physicians Evidence-Based Clinical Practice Guidelines comprise strategies for the management of thrombosis in neonates and children and may serve as orientation in this matter [107]. The named guidelines recommend doses of 1 to 5 mg/kg of ASA per day in anti-platelet therapy in children [107]. Taking this into consideration, the daily dosage of ASA of 2–3 mg/kg as suggested by Harrison et al. may seem appropriate for prophylactic treatment in childhood primary thrombocytosis [90]. T. Barbui had previously emphasized that anti-platelet therapy in patients with extreme thrombocytosis (> 1000 × 10^9^/l) should only be initiated in the presence of a ristocetin cofactor activity > 30% [108]. Furthermore, signs of abnormal bleeding should prompt screening for AvWS by determining both vWF antigen and a parameter reflecting vWF function such as ristocetin cofactor activity irrespective of platelet counts [11, 109]. In parallel to current guidelines on adult risk-adapted therapy [11], the recommendation on ASA-therapy by Kucine et al. may be extended to asymptomatic children with *JAK2*-V617F (or possibly other activating *JAK2* mutations), because this mutation is considered an independent risk factor for thromboembolic events in adults [93]. With regard to children, it has to be emphasized that within the study collective described by Randi et al., the three children affected by severe thromboembolic events all harbored *JAK2*-V617F [12]. Of note, in case of thrombotic complications, treatment with ASA alone may not be sufficient.

### When to consider cytoreductive treatment in children?

Because of the potential side effects, the ELN advocated a restrictive use of cytoreductive therapy in pediatric thrombocytosis [90, 96, 108]. Kucine et al. proposed cytoreductive treatment for the ET high-risk group comprising children who failed low-risk therapy and those with a history of thrombosis or severe hemorrhage [1]. Furthermore, cytoreductive therapy was recommended in children with persistent extreme thrombocytosis [1]. However, in adults, ELN recommendations only suggest cytoreductive treatment if platelet counts exceed 1500 × 10^9^/l [96], and Tefferi et Barbui underlined that extreme thrombocytosis alone does not suffice to indicate cytoreductive treatment [11]. This highlights the difficulties in appropriately determining indications for cytoreductive treatment in children with primary thrombocytosis.

Furthermore, the choice of agent is especially difficult at childhood age. In parallel to adult guidelines, Kucine et al. suggested hydroxyurea as possible first-line therapy should cytoreductive treatment be necessary in childhood ET [1]. Due to the possibility of increased malignant transformation and secondary malignancies, the use of hydroxyurea in children was controversially discussed in the past [1, 102]. Still, it is important to note that in children and even infants with sickle cell disease, hydroxyurea is now regularly used as a long-term therapy due to its effectiveness in preventing severe complications of the disease [110]. Current evidence including this long-term experience in children does not support concerns about leukemogenicity [11, 110]. However, hydroxyurea may cause male infertility, which is why fertility- preserving measures should be initiated before treatment with this drug is started in the young [110].

As the use of interferon-alpha (IFN) or pegylated interferon-alpha (PEG-IFN) has been suggested in younger adults and pregnant women, these agents may constitute a preferred treatment option in children [11, 96]. However, common side effects of IFN including influenza-like as well as neuro-psychiatric symptoms and the need of subcutaneous application may complicate the adherence to treatment particularly in younger children [99].

Anagrelide constitutes a further non-leukemogenic treatment option but experience in children is extremely limited, and in adults, a higher risk of fibrotic transformation, hemorrhagic events, and arterial embolism were reported in patients treated with anagrelide as compared to those who received hydroxyurea [101, 104].

Other agents such as busulfan are only occasionally used as second-line therapy in elderly adults due to concerns about leukemogenicity and therefore are clearly not indicated for the treatment of thrombocytosis in children [11, 96].

In principle, currently suggested thresholds and treatment strategies need validation by prospective data specifically acquired in childhood ET. However, it is most challenging or virtually impossible to obtain such data because of the rarity of primary thrombocytosis and the necessary long follow-up times. The use of international registries may thus serve as a second-best alternative to obtain clinically useful data. For the time being, the use of cytoreductive therapy in childhood thrombocytosis should remain as restrictive as possible.

### Are recommendations the same in childhood hereditary thrombocytosis?

In the past, several authors have argued for a different management of primary hereditary versus acquired thrombocytosis. Thus, in children with primary hereditary thrombocytosis, Giona et al. recommended to merely monitor asymptomatic patients and to use ASA only when microcirculatory symptoms or thromboembolic complications occur [106]. These recommendations were based on a study group of 16 children with primary hereditary thrombocytosis of which 15 harbored an *MPL* mutation at codon 505 [106]. Of note, *MPL*-S505N was previously described to convey a high risk of thromboembolic events [45]. In the treatment algorithm by Giona et al., cytoreductive therapy was not included among the standard treatment options in childhood hereditary thrombocytosis, however considered in acquired primary thrombocytosis in case of resistance to ASA or progressive organomegaly [106]. Tefferi et al. equally argued for an especially restrictive use of cytoreductive treatment in children with hereditary thrombocytosis while relying on anti-platelet therapy [23].

## Discussion

When classifying childhood thrombocytosis, the high proportion of children with acquired primary thrombocytosis but triple-negative mutation status for the known driver mutations is striking. This was highlighted both in single studies [12] as well as in a recent review by Ianotto et al. [78]. As the latter pointed out, some of the triple-negative patients might represent misdiagnosed cases of secondary thrombocytosis [78]. As a further explanation, Randi et al. postulated that some of these cases might not suffer from true myeloproliferative disease but that non-clonal mechanisms may be responsible for increased thrombocytosis in these patients [12]. This would have immediate consequences on the clinical management of these children, and the authors demanded longer clinical monitoring and a restrictive use of cytoreductive treatment in such cases [12]. As a second major issue, the so-called fraction of “familial MPN” is still difficult to classify as the postulated underlying mutations remain largely unknown. It was suggested by Olcaydu et al. that mutations in these patients predispose for the acquisition of somatic mutations that may enhance the risk of neoplastic transformation to MPN or other cancers [59].

When diagnosing chronic thrombocytosis in children, the distinction between primary and secondary forms may be challenging. The interval of re-evaluation of platelet counts may be crucial. While early re-evaluation might prompt unnecessary invasive diagnostic procedures in children with persistent secondary thrombocytosis, choosing an interval too long might entail late diagnosis of myeloid malignancies such as Philadelphia-positive CML or MPNs that require treatment. Kucine et al. suggested an interval of 3–4 weeks before re-evaluating platelet counts [1]. While this seems appropriate it has to be kept in mind that the suggested algorithm has not been validated on larger patient cohorts, and individual adjustments may be necessary. When secondary thrombocytosis is diagnosed, the platelet count should be re-evaluated when the primary cause has been successfully treated. As for other rare diseases, the choice of the methodology for genetic testing and in particular the use of unbiased genomic analyses rather than methods targeting single genes has been evolving with the reduction of cost of genome analysis. However, it needs to be considered that such analyses must be preceded and paralleled by professional genetic counseling. The present algorithm thus proposes a two-step screening approach with priority to known MPN genes.

The most important questions arise from current management of childhood thrombocytosis. Of note, the suggested treatment recommendations all require validation and are mostly based on case descriptions and on guidelines for adults. As a major aim, treatment should reduce the risk of thromboembolic complications in primary thrombocytosis while limiting the side effects of prophylactic interventions. However, these risks can only be estimated with a high degree of uncertainty. The fact that there are substantial uncertainties about the correct classification and diagnosis of pediatric ET in published reports significantly adds to these difficulties [12, 78]. Thus, the risk of thrombotic complications in children, which was recently reported as only 3.8% in ET, may be underestimated if cases of secondary thrombocytosis are incorrectly diagnosed as ET [78]. As mentioned before, the use of international registries may be helpful to better define these risks and treatment effects in the future.

For the time being, the authors agreed that low-dose antiplatelet-therapy in pediatric ET should be started in case of symptoms (such as erythromelalgia, severe headaches, splenomegaly), in the presence of *JAK2*-mutations or other additional thrombophilic risk factors, and considered in case of persistent extremely elevated platelet counts (> 1000 × 10^9^/l). Obviously, this measure needs to be paralleled by monitoring for most importantly AvWS but also Reye syndrome and by careful education of families regarding clinical signs of these complications. In case of thrombotic complications, adequate thrombosis management and prophylactic treatment should be initiated, and ASA therapy alone may not suffice. In line with previous recommendations, the group agreed that cytoreductive treatment should only be used as an exception and mainly be considered in case of major thrombotic or bleeding complications [1, 108]. If necessary, PEG-IFN as a non-leukemogenic treatment option may be used in children. Furthermore, with increasing data on the long-term use in children, hydroxyurea seems to be an additional safe treatment option if fertility-preserving measures are taken into consideration.

### Conclusions

Primary thrombocytosis in children is rare compared to secondary forms. Important triggers of secondary thrombocytosis include infection and inflammation as well as iron deficiency, which may be identified through clinical examination and basic laboratory assessment. In case of persistent childhood thrombocytosis without secondary stimuli, both myeloproliferative neoplasms and hereditary primary thrombocytosis have to be considered. In this case, the diagnostic assessment should include the search for myeloid malignancies and their causative somatic mutations, as well as the search for rare germline mutation causing hereditary thrombocytosis. Monitoring alone is likely appropriate in asymptomatic, mild forms of chronic thrombocytosis. Symptomatic children or those with additional risk factors such as presence of *JAK2*-V617F may benefit from therapy with low-dose ASA along with careful monitoring while keeping in mind the risk of acquired von Willebrand syndrome and potentially Reye syndrome. Cytoreductive treatment may rarely be required as an escalation in case of non-controlled vascular complications. As prospective, large-cohort data are needed in order to validate the current suggested recommendations, it is recommended to include patients in international registries.

## Data Availability

With few selected exceptions, included references are available on the Pubmed database.
